# Ultrasound-Guided Percutaneous Placement of Indocyanine Green-Dyed Coils for Intraoperative Localization of Subpleural Lung Tumors

**DOI:** 10.7759/cureus.119258

**Published:** 2026-11-07

**Authors:** Sammy Onyancha, Hasnain Bawaadam, Peter Hollaus, Gernot Rohde, Waldemar Schreiner

**Affiliations:** 1 Pulmonology, St. Elisabethen Krankenhaus, Frankfurt, DEU; 2 Critical Care, Aurora Medical Center, Kenosha, USA; 3 Thoracic Surgery, St. Elisabethen Krankenhaus, Frankfurt, DEU; 4 Pulmonology, Universities of Giessen and Marburg Lung Center, Marburg, DEU

**Keywords:** fiducial maker, icg, indocyanine green (icg), intraoperative icg, intraoperative localization, lung-sparing sublobar resection, sublobar resection, transthoracic ultrasound, ultrasound-guidance, video-assisted thoracoscopic surgery

## Abstract

Accurate localization of peripheral pulmonary lesions is a prerequisite for lung-sparing minimally invasive resection. Indocyanine green (ICG)-dyed coils have emerged as an effective multimodal marker, detectable by near-infrared fluorescence, fluoroscopy, palpation, and ultrasound, but their placement currently relies on CT or cone-beam CT guidance, with the associated radiation exposure and logistical demands. For subpleural lesions, transthoracic ultrasound offers real-time, radiation-free guidance. We present our initial experience with ultrasound-guided percutaneous placement of ICG-dyed coils.

Of 33 patients scheduled for ICG-coil marking between July 2025 and March 2026, eight were assessed for ultrasound-guided marking, and three (37.5% of those assessed; 9% of all scheduled) had a lesion with a suitable acoustic window. In these three patients (aged 52-67 years), an ICG-dyed coil was placed adjacent to the tumor under real-time transthoracic ultrasound guidance in the operating room under general anaesthesia, immediately before uniportal or biportal video-assisted thoracoscopic surgery (VATS). The lesions measured 17-19 mm and were located 2-4 mm below the visceral pleura in the left upper, right lower and right upper lobe. Coil placement was technically successful in all patients, with a median procedure time (first ultrasound contact to needle removal) of 12 minutes (range 11-13 minutes) and a median total added time, including preparation, of 33 minutes. The marker was identified by near-infrared fluorescence in every case, and all lesions were resected with R0 margins. Histology showed squamous cell carcinoma in two patients and metastatic adenocarcinoma of colorectal origin in one. No bleeding or other immediate complications were observed at thoracoscopy. Because the lung was collapsed for resection immediately after puncture, delayed pneumothorax and marker migration could not be assessed.

In selected patients with solid subpleural lesions and an adequate acoustic window, ultrasound-guided placement of ICG-dyed coils was technically feasible and enabled fluorescence-guided localization at thoracoscopy. However, due to the small series, the safety of the technique or its added value over intraoperative palpation or thoracoscopic ultrasound cannot be established. Larger prospective studies, including marking with delayed resection, are needed to define patient selection and to compare the technique with CT-guided and bronchoscopic marking.

## Introduction

Minimally invasive thoracic surgery has become the standard approach for the management of early-stage lung cancer and selected pulmonary metastases [1]. With the growing detection of small pulmonary lesions and the increasing adoption of lung-sparing sublobar resection [2,3], accurate intraoperative identification of the target has become a central technical challenge. Small, deep or part-solid nodules are often neither visible on the pleural surface nor palpable, and the limited tactile feedback inherent to uniportal video-assisted techniques may result in prolonged operating times, inadequate margins or conversion to thoracotomy [4].

To overcome this limitation, several preoperative localization methods have been developed, including hookwires, microcoils, dyes, radiotracers and fiducial markers, most commonly placed under CT guidance [5-7]. Each of these has notable limitations. Hookwires may dislodge and require surgery shortly after placement, liquid dyes diffuse over time, and radiotracer techniques depend on nuclear medicine infrastructure [7-9].

Indocyanine green (ICG) is a fluorescent dye that absorbs near-infrared light and emits fluorescence at a slightly longer near-infrared wavelength. Because near-infrared light penetrates tissue considerably better than visible light, a near-infrared thoracoscopic camera can detect ICG through the visceral pleura and a thin layer of overlying lung, thus providing real-time visual guidance. ICG is therefore increasingly used for pulmonary nodule localization [10]. When injected as a liquid, however, the dye diffuses over time. More recently, ICG-dyed embolization coils have been introduced. A fibred metallic embolization coil is soaked in ICG solution before placement, so that the dye is retained on the coil fibres and produces a focal fluorescent signal at the marker site, combining the visual signal of the dye with a stable physical marker [11]. These coils offer multimodal detectability: fluorescence under near-infrared imaging, radiopacity for fluoroscopic visualization, tactile feedback during palpation, and hyperechogenicity on ultrasound [12]. Because the marker remains in place, marking and surgery can in principle be separated in time, allowing more flexible scheduling [11]. This potential advantage was not tested in the present series, in which marking was performed immediately before resection

Placement of ICG-dyed coils has been described percutaneously under CT guidance and bronchoscopically under cone-beam CT guidance, including without robotic assistance [13-15]. Both approaches, however, expose patients and staff to ionizing radiation and depend on access to a CT scanner or hybrid suite.

Transthoracic ultrasound is an established, radiation-free modality for real-time guided biopsy of pleural-based and subpleural lesions [16]. This raises the possibility of using ultrasound alone to guide marker placement in selected patients, particularly those with lesions abutting or lying immediately beneath the pleura and accessible through an intercoastal acoustic window. In this clinical subgroup, ultrasound guidance could potentially replace CT or cone-beam CT guidance, whereas deeper lesions would continue to require CT-guided or bronchoscopic marking.

In this report, we present a series of three patients scheduled for uniportal or biportal VATS who underwent ultrasound-guided percutaneous placement of ICG-dyed coils for subpleural lesions, with the aim of assessing the feasibility of the technique. These cases also illustrate the key considerations for patient selection and workflow.

## Technical report

Study design and patient selection

This retrospective single-centre case series describes three patients with peripheral pulmonary lesions scheduled for minimally invasive resection who underwent ultrasound-guided tumor marking with ICG-dyed coils at St. Elisabethen Krankenhaus, Frankfurt, Germany, between July 2025 and March 2026. The objective was to assess technical feasibility and immediate procedural safety; owing to the descriptive design and small cohort, no hypothesis testing was performed, and proportions are reported with exact (Clopper-Pearson) 95% confidence intervals (CI).

The indication for resection and marking was established by a multidisciplinary tumor board. During the study period, 33 patients were scheduled for preoperative lesion marking. Bronchoscopic marking was the institutional standard and was used directly in 25 of them, whose lesions had a positive bronchus sign and were located in the outer third of the lung but remained more than 1 cm from the pleural surface on CT. The remaining eight patients were assessed for percutaneous guided marking as part of the preoperative planning work-up because the lesion lay within 5mm of the lateral costal pleura on CT. On CT review, five of these were considered unsuitable because aerated lung tissue was interposed between the lesion and the potential puncture site, and they underwent bronchoscopic marking instead. The remaining three patients (3/8 assessed, 37.5%, 95% CI 8.5-75.5%; 3/33 scheduled, 9.1%, 95% CI 1.9-24.3%) underwent ultrasound-guided percutaneous marking and are the subjects of this report (Figure 1). 

**Figure 1:**
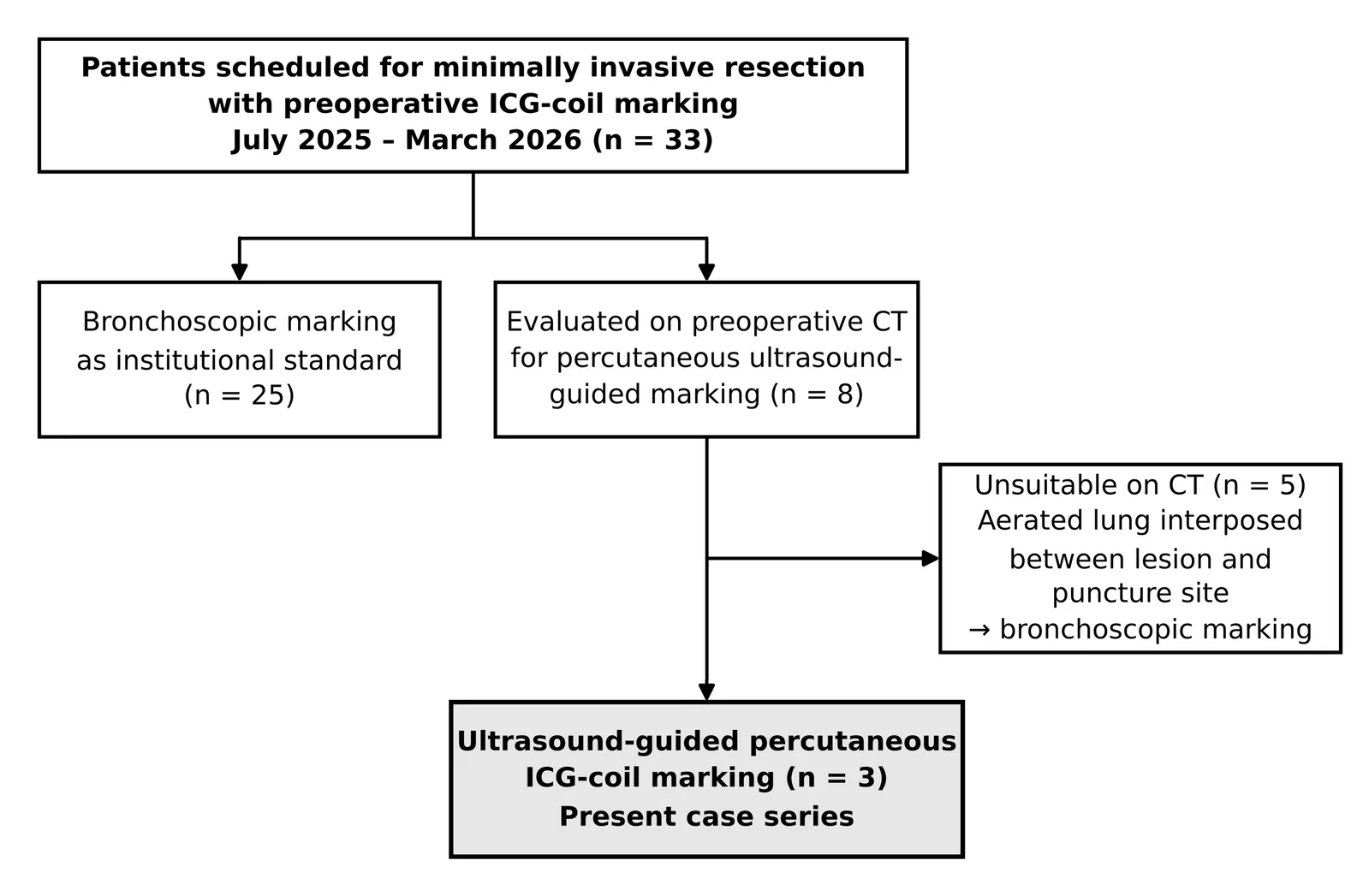
Patient selection flow chart ICG: indocyanine green

Patients were considered eligible for ultrasound-guided marking if the target lesion was located within the subpleural surface and had visible contact with the lateral costal pleura on preoperative CT. The lesion accessibility as well as needle trajectory through an intercoastal space was determined in the coronal plane as this best represented the lateral decubitus positioning in which the periprocedural ultrasound examination would be performed. A further prerequisite was unequivocal sonographic identification of the lesion through an intercoastal acoustic window. In all three patients, a marker was chosen because the lesion was not expected to be reliably visible or palpable at uniportal or biportal video-assisted thoracoscopic surgery (VATS) and because the resection required precise localization for margin planning. Thoracoscopic ultrasound was not used routinely at our centre.

Localization procedure

All procedures were performed with the patient in the lateral decubitus position in the operating room under general anaesthesia. Patients were intubated with a double-lumen tube, and the puncture was performed under two-lung ventilation, prior to commencement of the surgical resection so that the lesion could be visualized through the chest wall in the aerated lung. All punctures were performed by an interventional pulmonologist (S.O.) with extensive experience in thoracic ultrasound as well as ultrasound-guided transthoracic interventions. Ultrasound was performed with a convex probe (5-2 MHz, C60e, Micromaxx Ultrasound System, SonoSite Inc., Bothell, USA).

A 25 mg vial of ICG (Verdye, Diagnostic Green, Munich, Germany) was diluted with 10 ml of sterile saline, giving a final concentration of 2.5 mg/ml (Figure 2). 

**Figure 2:**
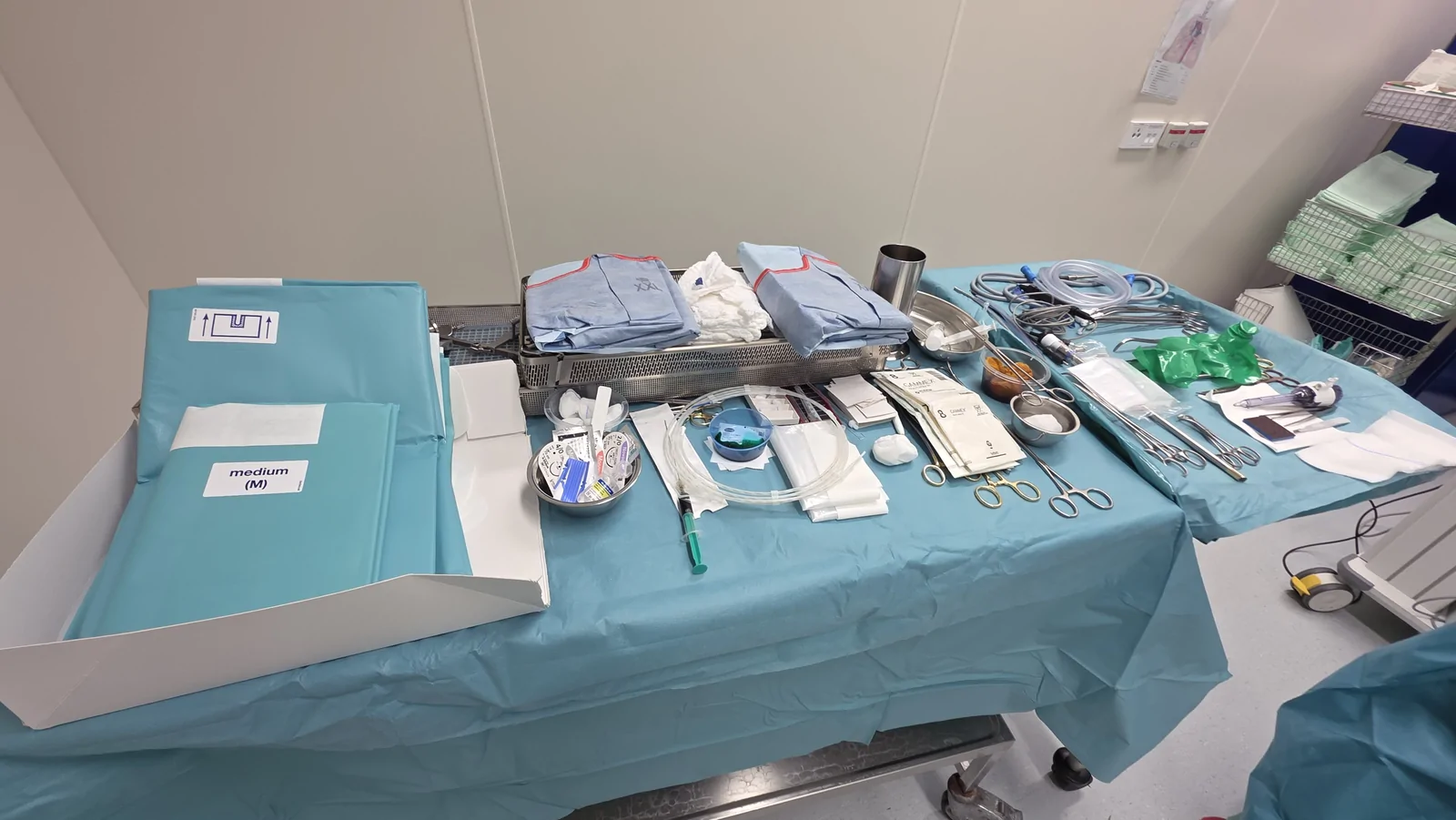
Sterile preparation of materials.

The target lesion was then identified by transthoracic ultrasound, and the puncture site and needle trajectory were chosen with reference to the preoperative CT. An embolization coil (MWCE-35-6/3 Tornado embolization coil, 0.035-inch, 6 mm to 3 mm coiled embolus diameter, 5.8 cm extended length, Cook Medical, Baesweiler, Germany) pre-soaked in 1 mL of the diluted ICG for 10 minutes (Figures 3, 4) was then deployed through a 15-gauge introducer needle (COX-2000-15-0101, Möller Medical GmbH, Fulda, Germany) using a 0.035 inch guidewire under continuous real-time ultrasound visualization (Figure 5). 

**Figure 3:**
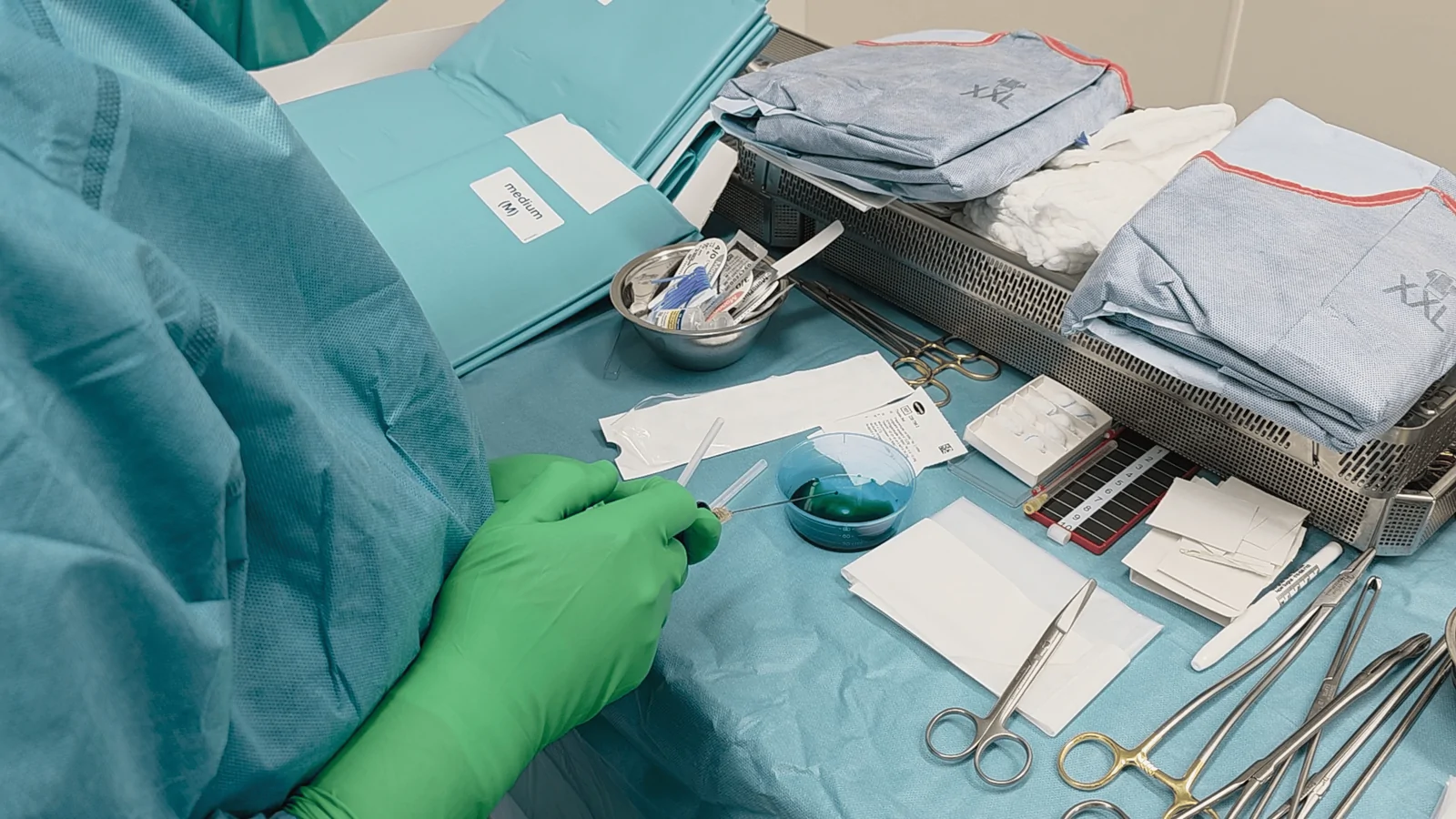
Pre-soaking the indocyanine green coil before placement.

**Figure 4:**
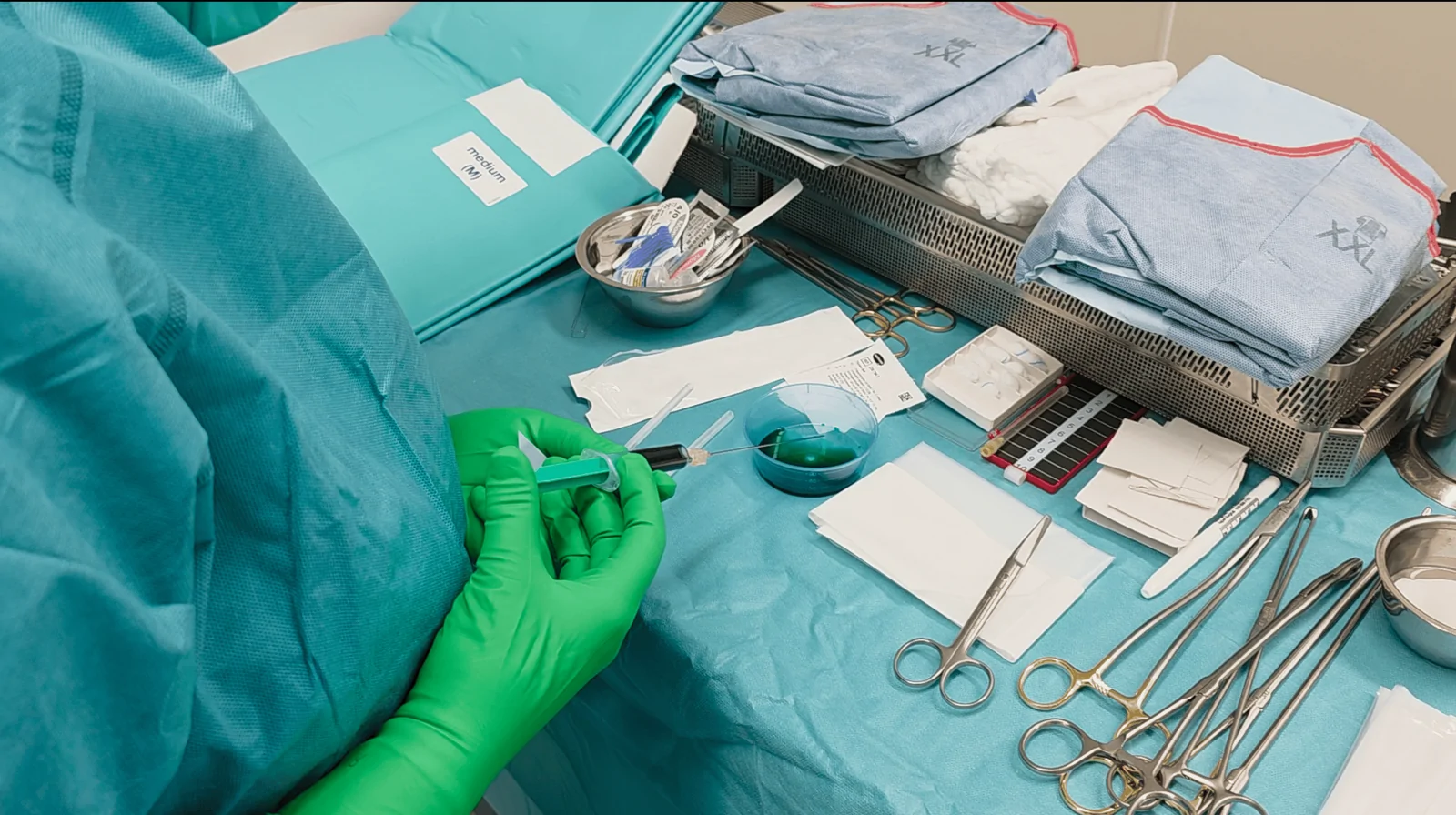
Pre-soaking the indocyanine green coil before placement.

**Figure 5:**
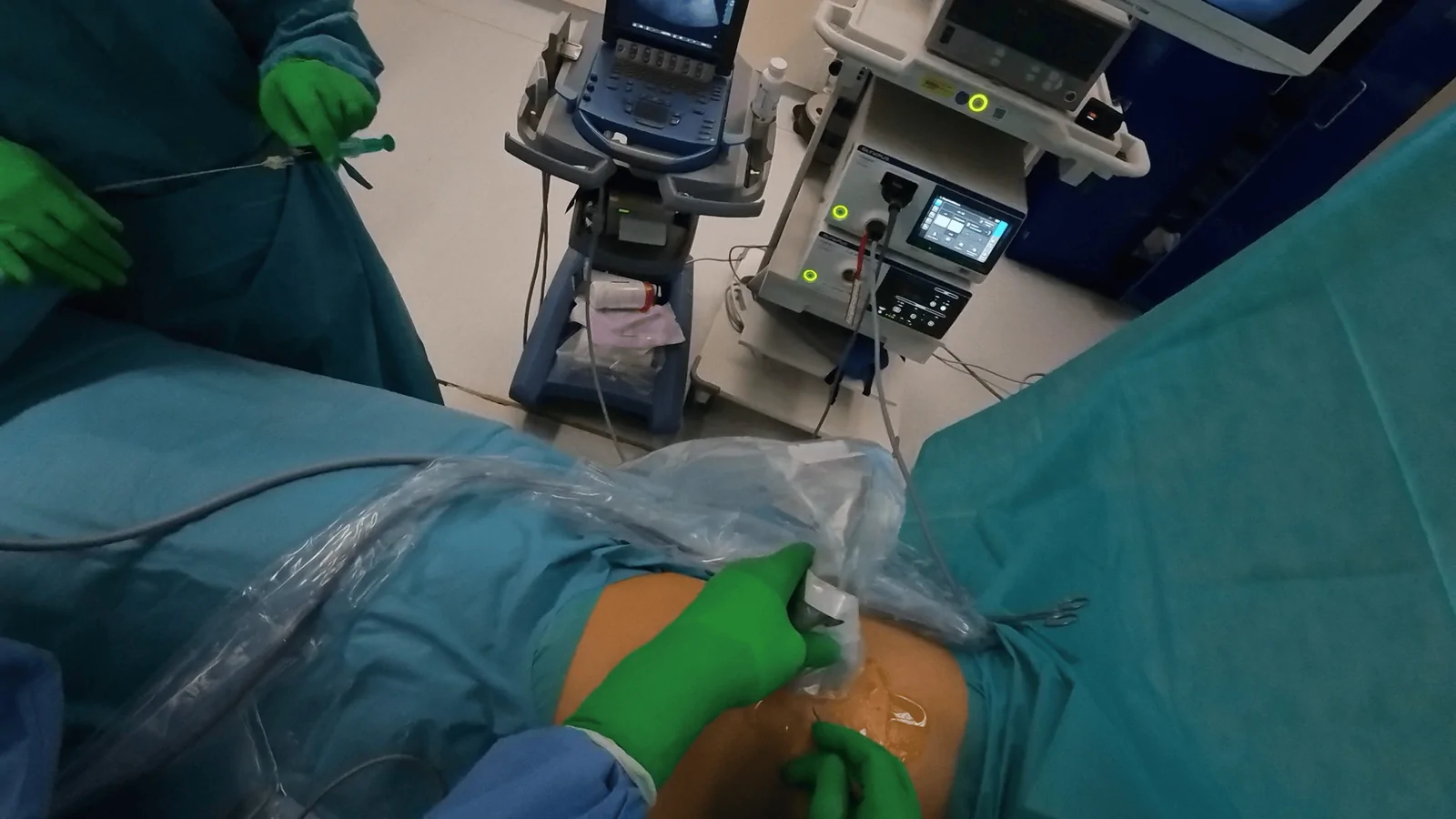
Needle puncture under sonographic guidance.

The coil was positioned adjacent to, rather than within, the tumor to avoid possible seeding. Correct coil position was confirmed sonographically by the hyperechoic appearance of the coil. Procedure time was recorded in the procedural records and was defined as the interval from first ultrasound contact to needle removal. In addition, the total added time before the start of resection, including ICG preparation, coil soaking and sterile set-up, was derived from the anaesthesia records. After needle removal, the puncture site was re-examined sonographically, and pneumothorax was excluded by the presence of lung sliding. One-lung ventilation with collapse of the ipsilateral lung was subsequently initiated and surgical resection commenced.

Surgical resection and outcome assessment

Resection was performed by uniportal or biportal VATS. At the start of thoracoscopy, the visceral pleura over the target area was inspected for visible changes, and the lesion was palpated as far as the access allowed. The coil was then localized intraoperatively by near-infrared fluorescence (Visera Elite II, OTV-S200, Olympus Medical Systems, Tokyo, Japan and Visera Elite III, OTV-S700, Olympus Medical Systems, Tokyo, Japan). The outcomes assessed were technical success of coil placement, procedure time, total added time, intraoperative identification of the marker, resection margin status, final histopathology, and procedure-related complications. Procedure-related complications were assessed at thoracoscopy (immediate pleural or parenchymal bleeding and ICG spillage into the pleural cavity) and during the postoperative course. The postoperative course was classified as uncomplicated if no complication of Clavien-Dindo grade I or higher, prolonged air leak (> 5 days), or readmission occurred. Chest drain duration and length of hospital stay were documented in the patient records, and the patients were followed up at the outpatient clinic two weeks after surgery. The coil-lesion distance was measured by the pathologist on a formalin-fixed specimen in the plane through the coil and the lesion, as the shortest distance between the coil and the tumor edge. Because resection followed immediately, delayed complications of the puncture, such as late pneumothorax or marker migration, could not be assessed.

Case 1

A 52-year-old male active smoker (42 pack-years) with a history of colon cancer presented with chest pain. Imaging revealed multiple peripheral lesions in the left upper lobe suspicious for metastasis (Figure 6). 

**Figure 6:**
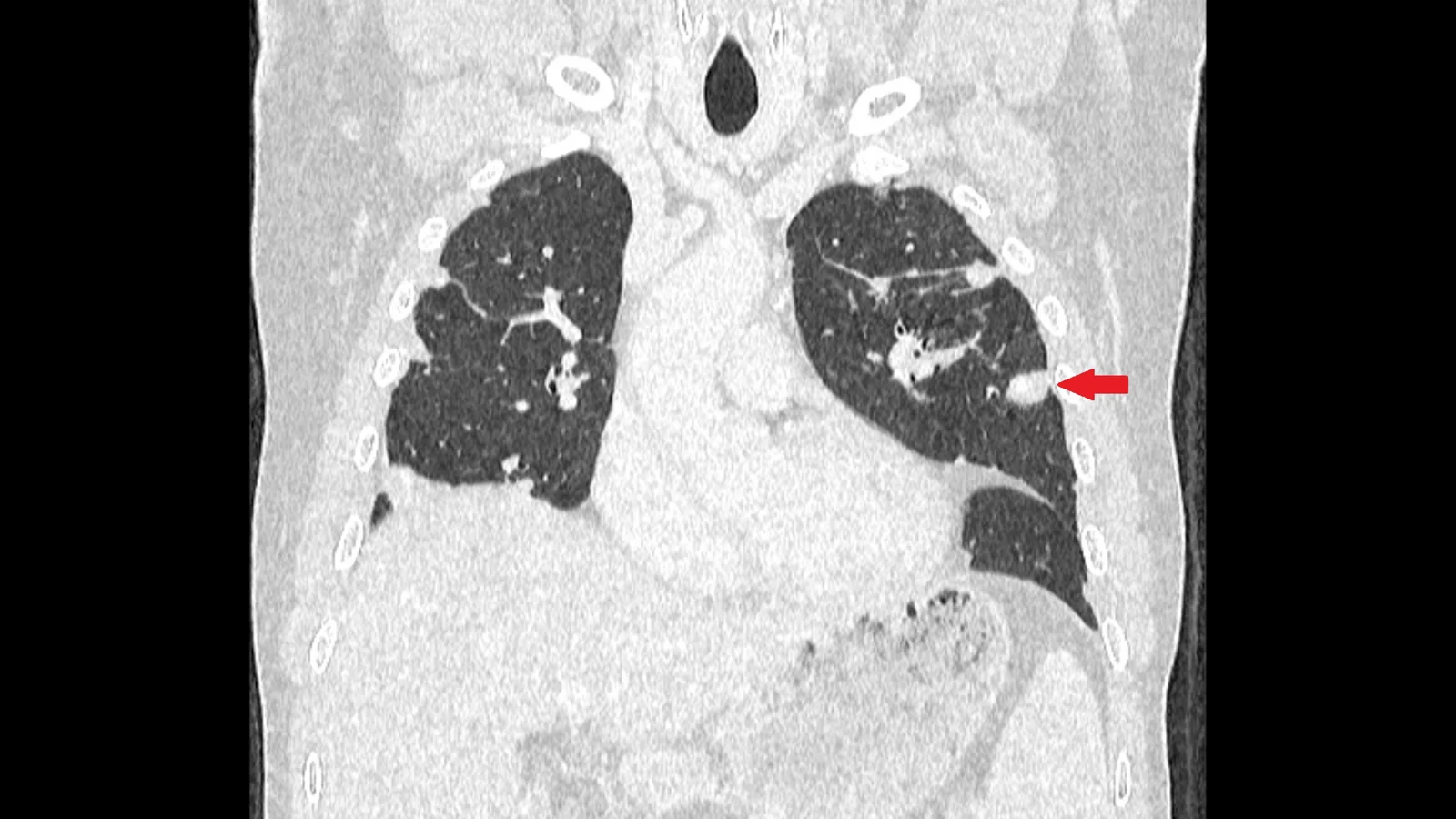
Chest CT demonstrating the peripheral lesions in the left upper lobe; a 17-mm lesion (red arrow) was selected for wedge resection.

A 17-mm lesion was clearly identified by transthoracic ultrasound, and an ICG-dyed coil was placed adjacent to it under real-time guidance (Video 1). 

**Video 1:** Ultrasound-guided deployment of the indocyanine green-dyed coil (Case 1).

The procedure took 13 minutes, and resection followed immediately. At uniportal VATS, the lesion was not palpable, and no changes were visible on the visceral pleura. The fluorescent marker was readily identified by near-infrared imaging (Figure 7), enabling localization and R0 wedge resection with a margin of 18mm (Figure 8). 

**Figure 7:**
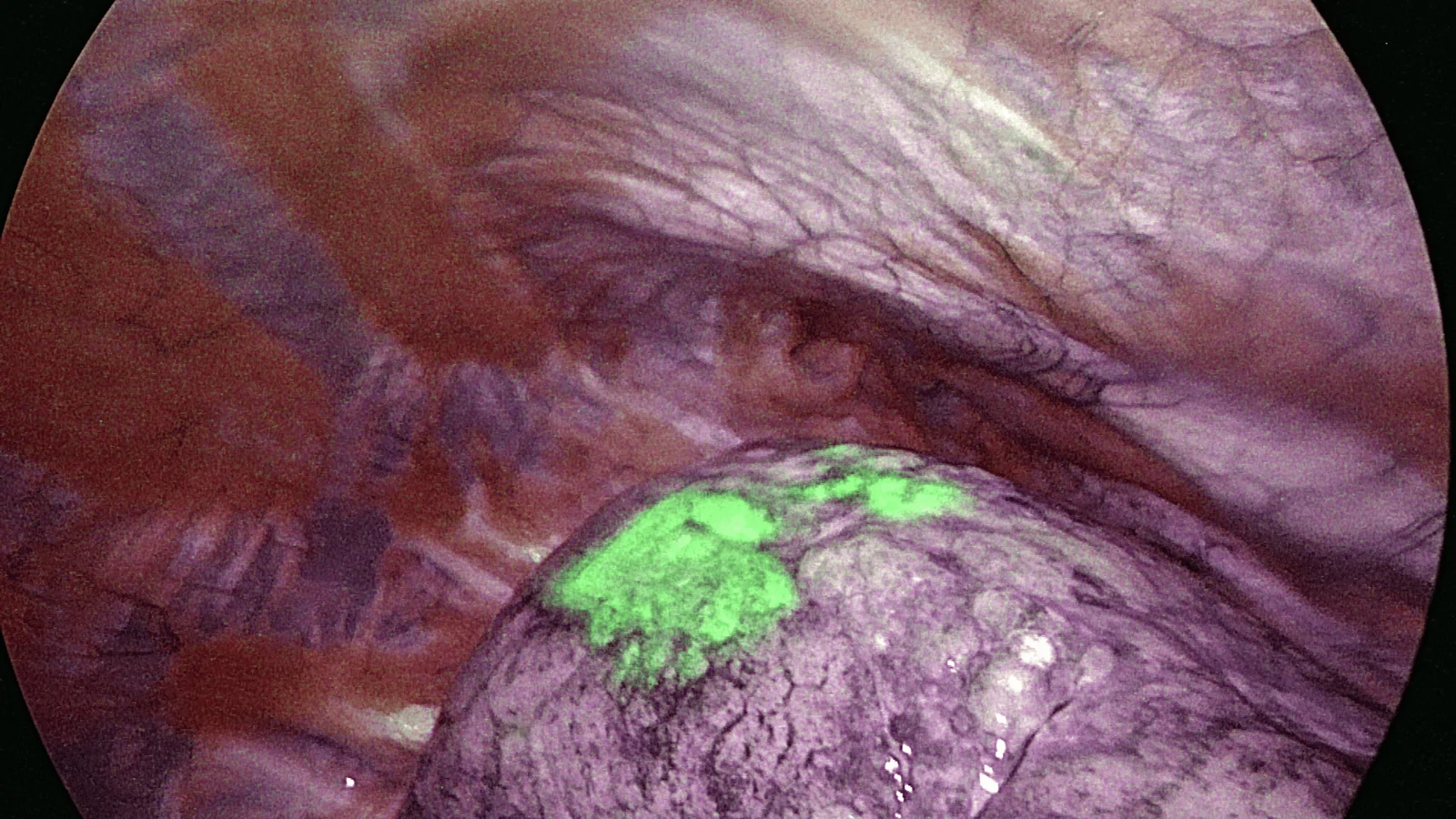
Intraoperative near-infrared view showing the fluorescent signal of the indocyanine green-dyed coil.

**Figure 8:**
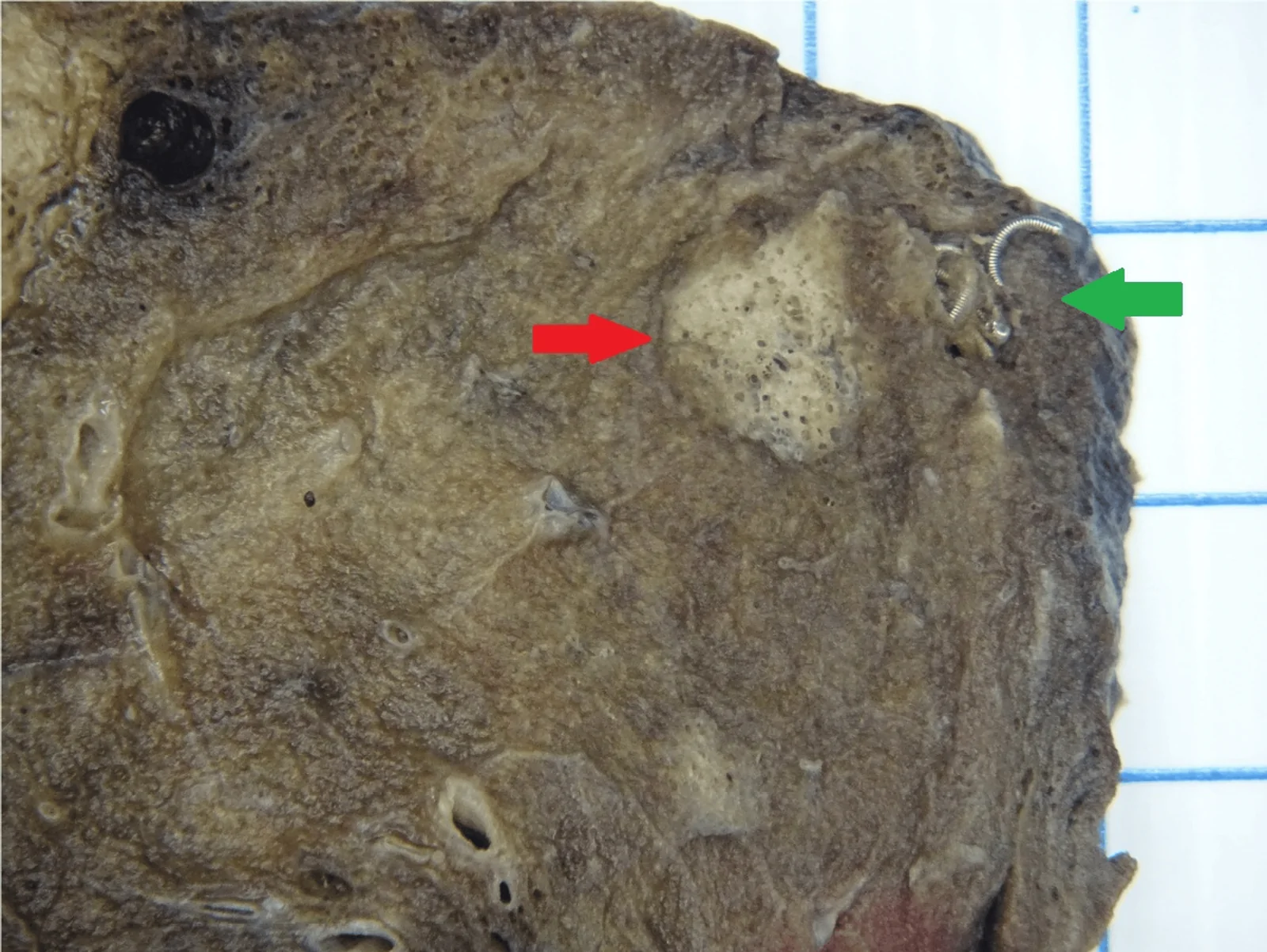
Wedge resection specimen containing the coil (green arrow) and lesion (red arrow).

Histopathology confirmed metastatic adenocarcinoma consistent with colorectal origin. The postoperative course was uncomplicated; the chest drain was removed on postoperative day 2, and the patient was discharged on day 4, and no complications were noted at follow-up on day 16.

Case 2

A 62-year-old female never-smoker was found to have an incidental 19-mm lesion in the right lower lobe on CT (Figure 9). 

**Figure 9:**
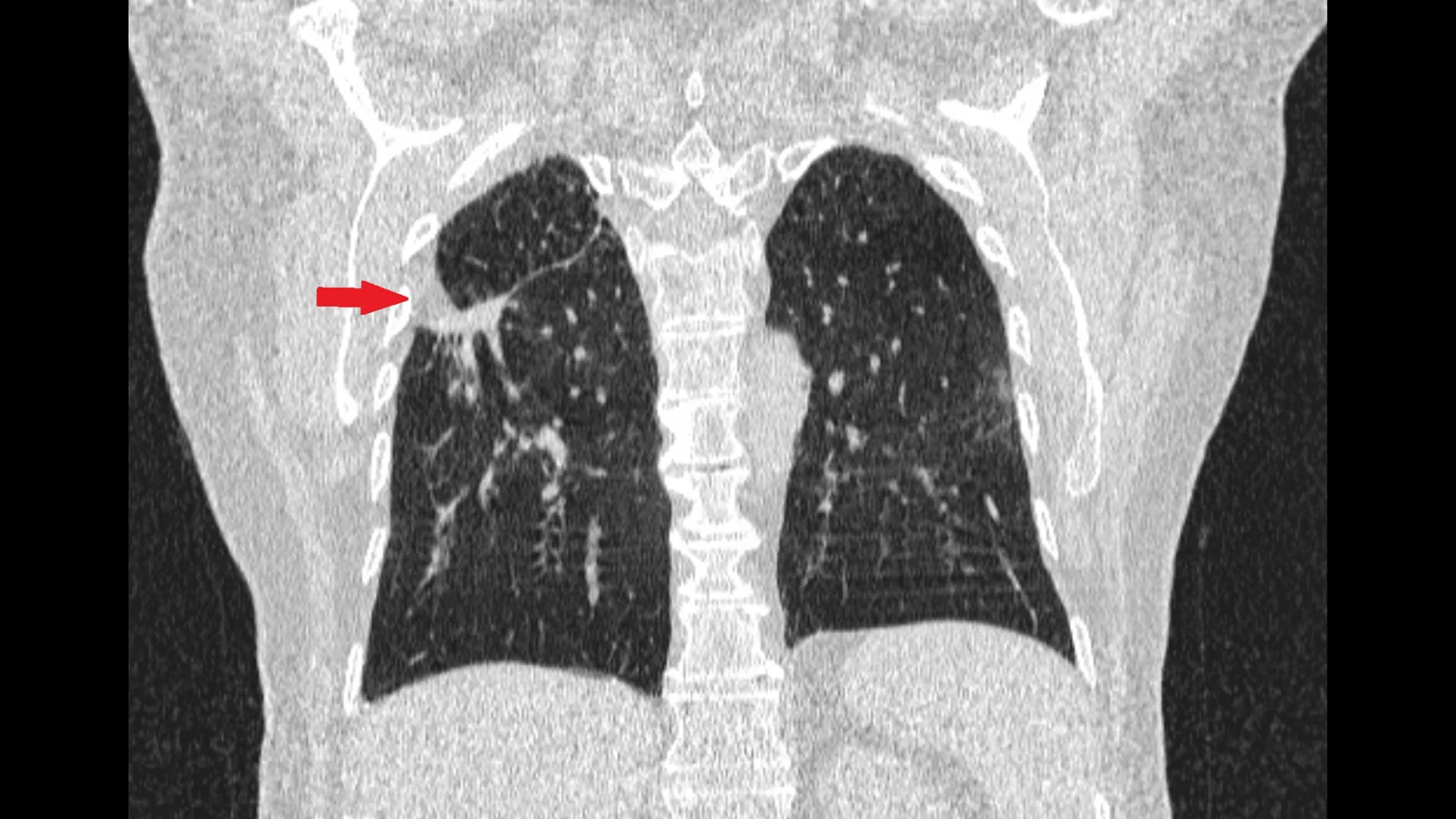
Chest CT demonstrating the peripheral lesion in the right lower lobe.

Ultrasound-guided placement of an ICG-dyed coil was completed in 11 minutes. At biportal VATS, the lesion was not palpable, and no changes were visible on the visceral pleura. The coil had remained in position, and the lesion was localized by fluorescence (Figure 10). 

**Figure 10:**
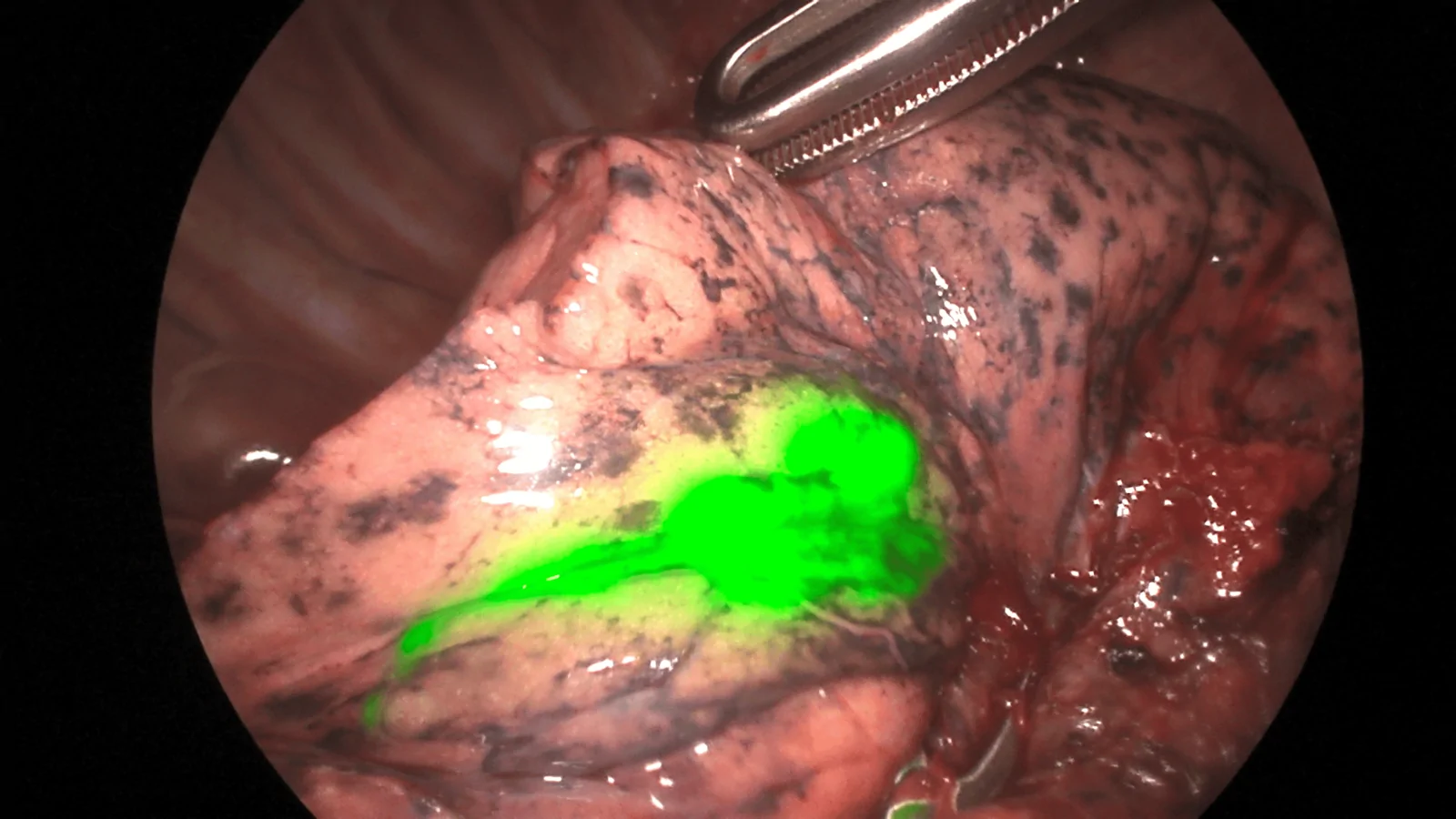
Near-infrared fluorescence of the indocyanine green-dyed coil.

R0 segmentectomy was achieved with a margin of 21 mm (Figure 11).

**Figure 11:**
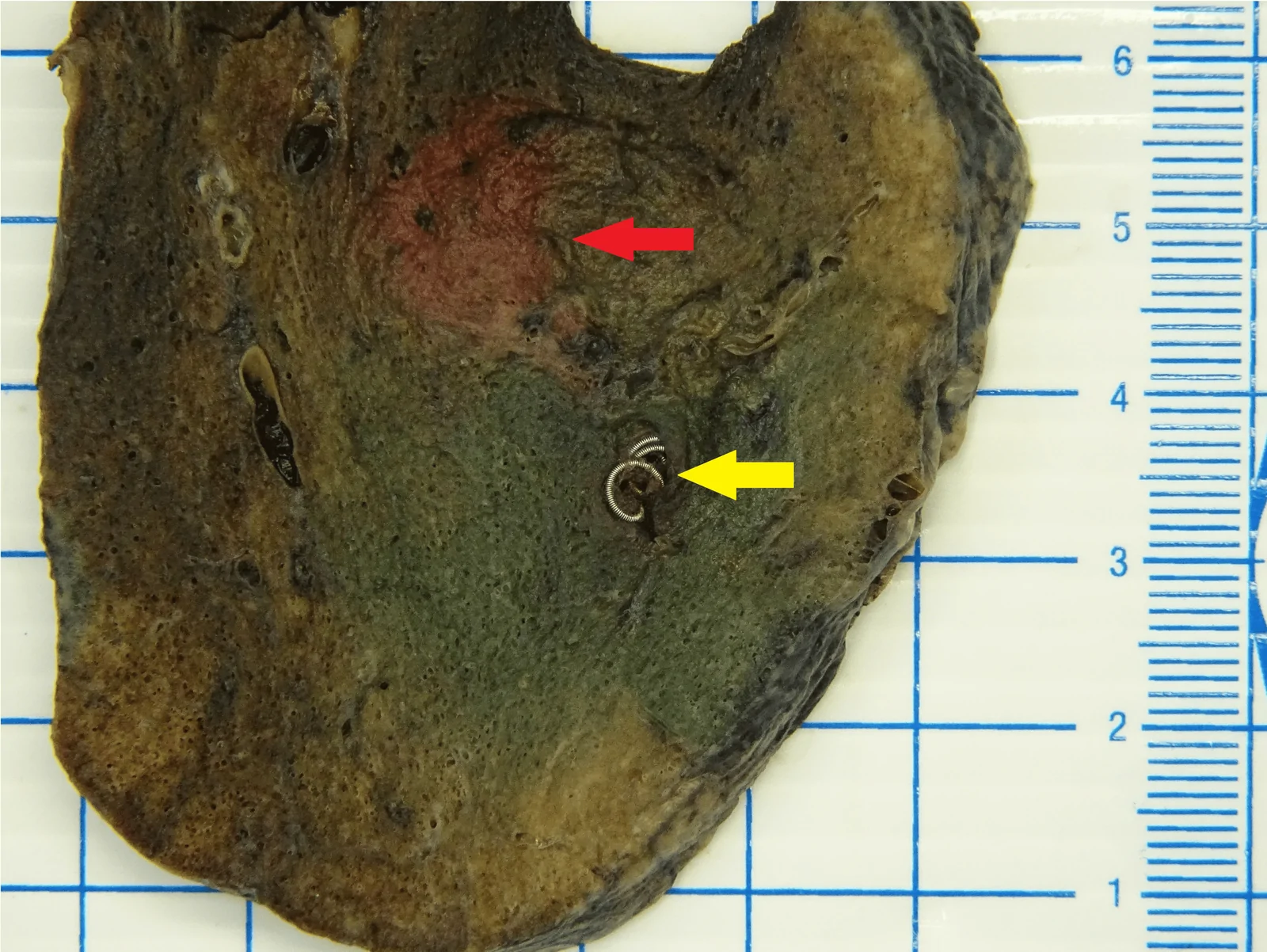
Segementectomy specimen containing the coil (yellow arrow) and tumor (red arrow).

Final pathology demonstrated squamous cell carcinoma. The postoperative course was uncomplicated, with the chest drain removed on postoperative day 3 and patient discharge on day 6. No further complications were noted on follow-up on postoperative day 18.

Case 3

A 67-year-old male ex-smoker (30 pack-years) presented with fever and a persistent cough of six weeks’ duration. Imaging identified an 18 mm peripheral lesion in the right upper lobe (Figure 12), and resection followed immediately.

**Figure 12:**
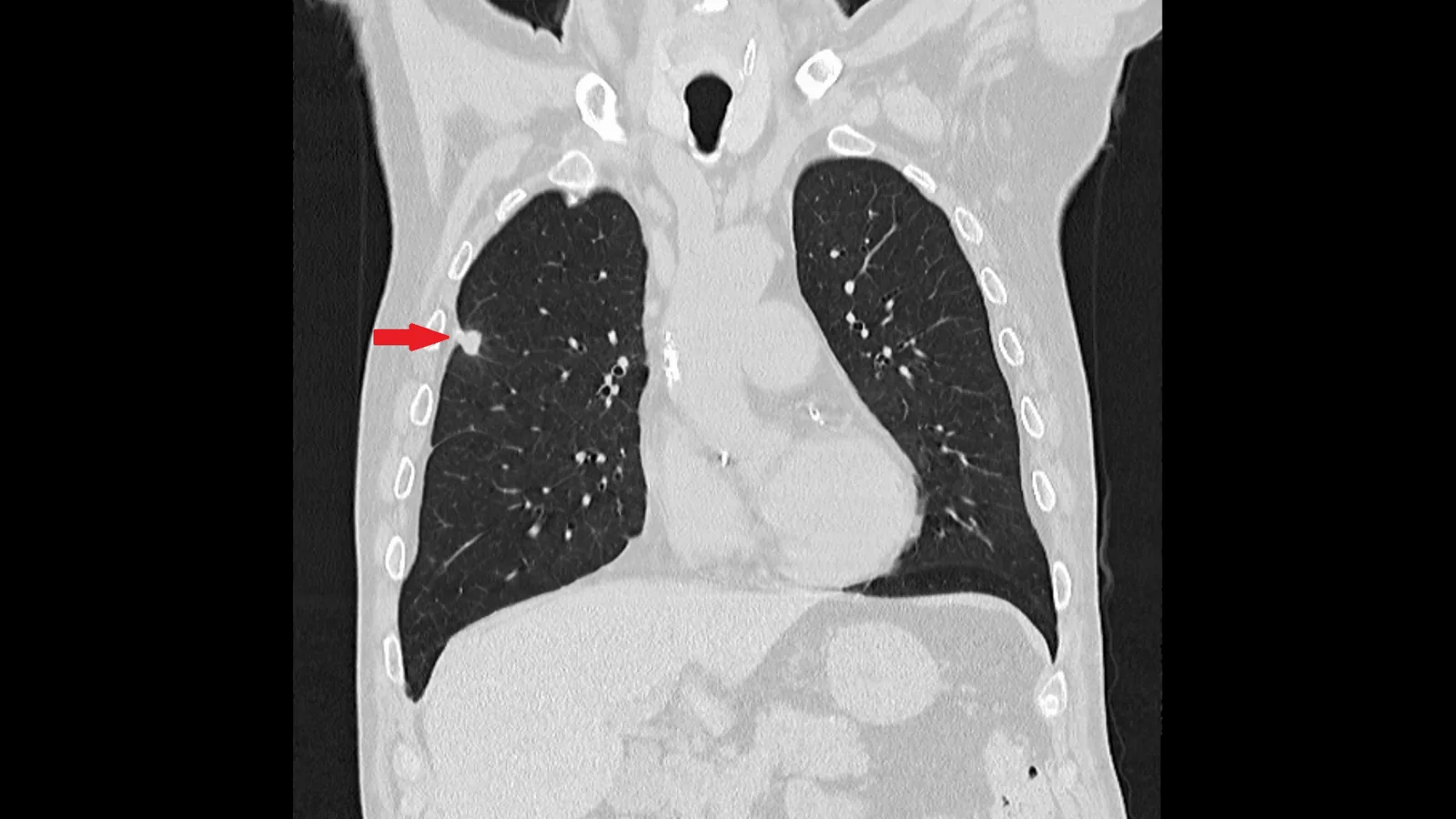
Chest CT demonstrating the peripheral lesion in the right upper lobe.

Ultrasound-guided coil placement was completed in 12 minutes (Video 2). 

**Video 2:** Ultrasound-guided deployment of the indocyanine green-dyed coil (Case 3).

At uniportal VATS, the lesion was not palpable, but light anthracotic changes were visible on the visceral pleura. Near-infrared fluorescence of the coil confirmed that these changes corresponded to the location of the lesion (Figure 13), and an R0 segmentectomy was performed with a margin of 22 mm (Figure 14). 

**Figure 13:**
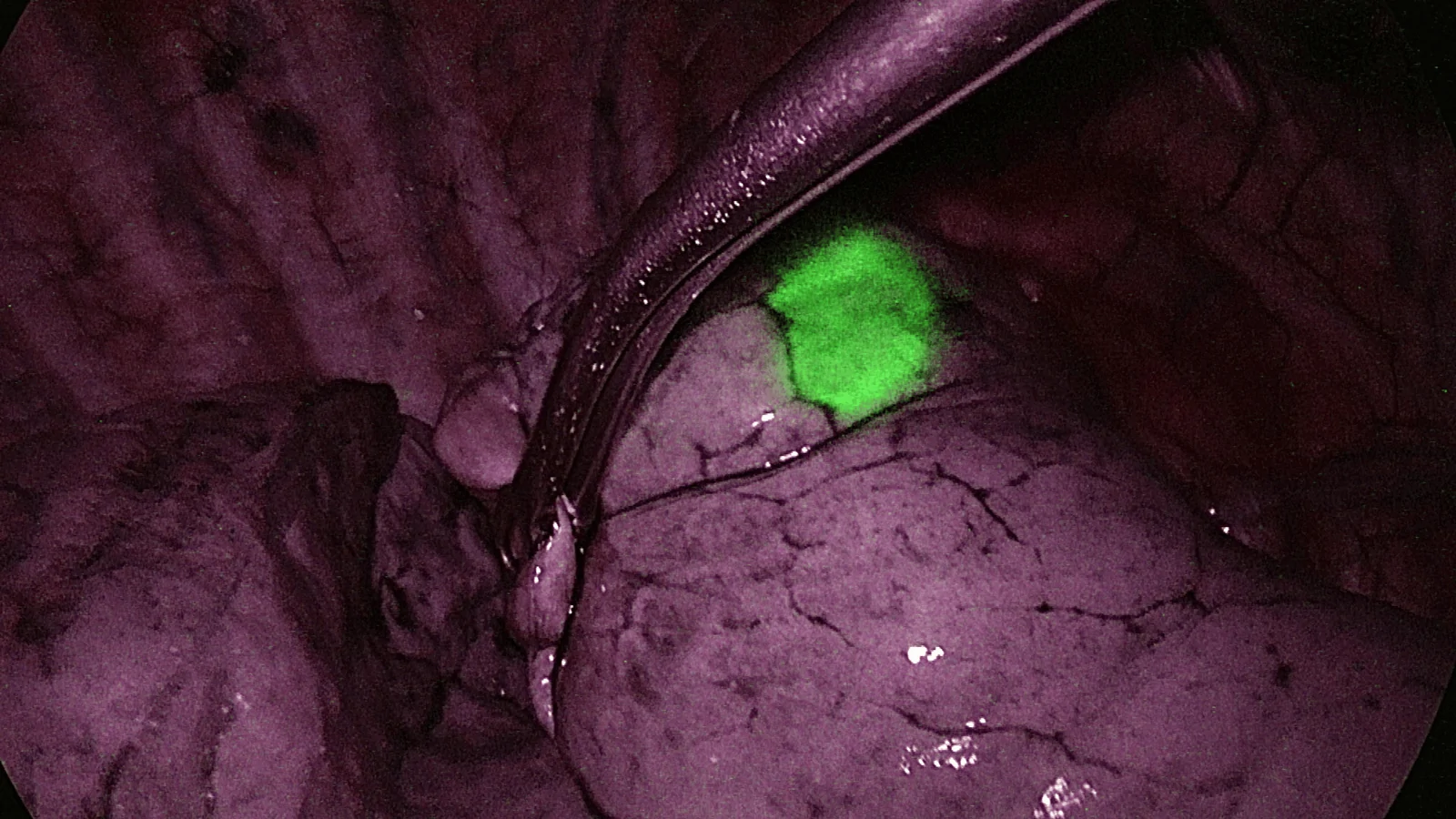
Intraoperative fluorescence of the implanted indocyanine green coil.

**Figure 14:**
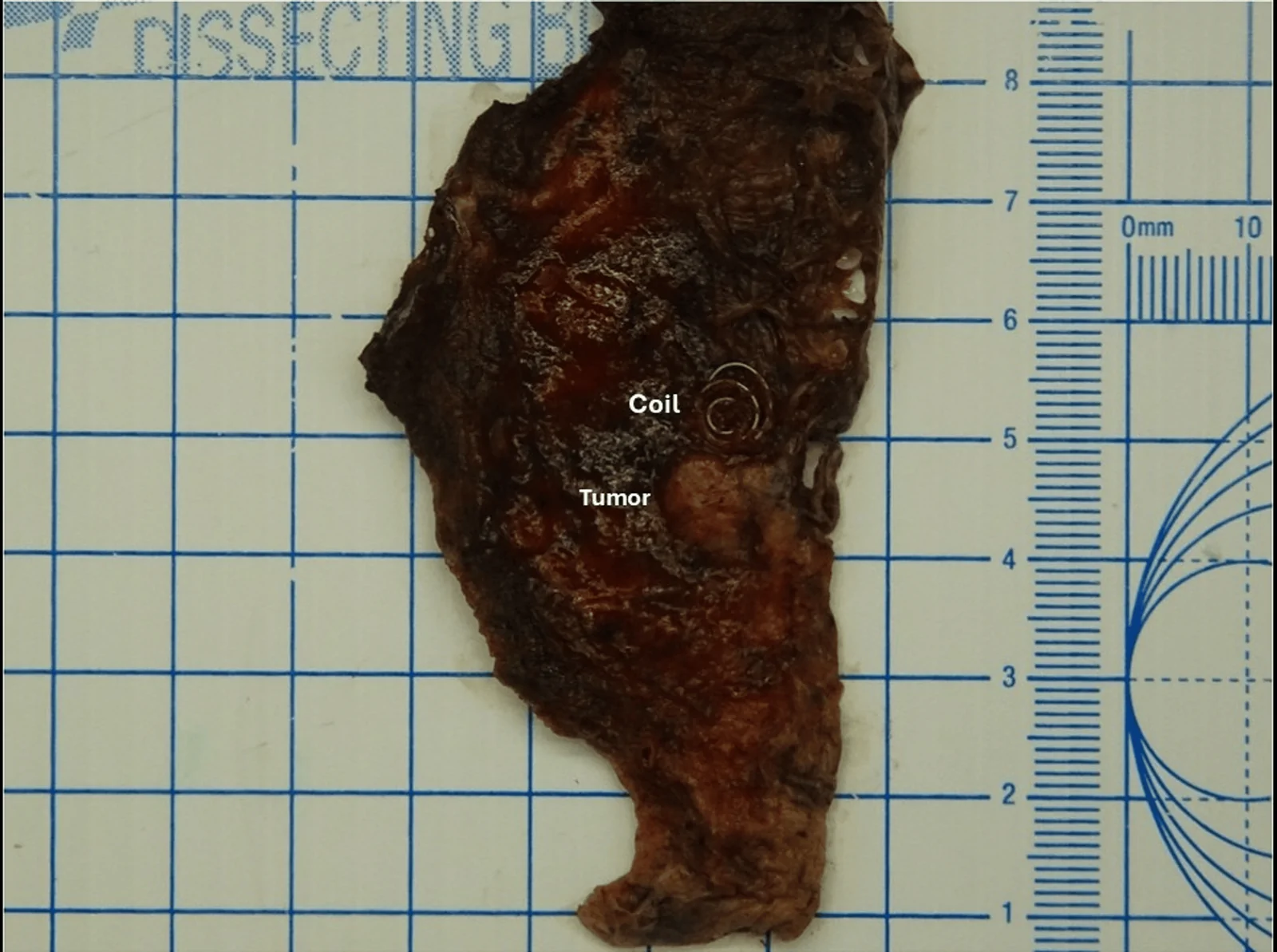
Resection specimen containing the coil and tumor.

Histology confirmed squamous cell carcinoma. The postoperative course was uncomplicated. The chest drain was removed on postoperative day 3, and the patient was released a day later. Follow-up on postoperative day 17 revealed no further complications.

Summary of outcomes

Coil placement was technically successful in all three patients (3/3; 95% CI 29.2-100%), with a median procedure time of 12 minutes (range 11-13 minutes) and a median total added time of 33 minutes (range 31-35 minutes). The lesions measured 17-19 mm. Lung sliding was preserved after needle removal in all patients. None of the lesions was palpable during uniportal or biportal VATS. In two patients (Cases 1 and 2), no changes were visible on the visceral pleura, and fluorescence of the coil was the only means of intraoperative localization. In the third (Case 3), fluorescence confirmed that light anthracotic pleural changes corresponded to the lesion. The marker was identified by near-infrared fluorescence in every case, and all lesions were resected with R0 margins (margin distance 18-22 mm). Coil-lesion distance, measured on the resected specimen, was 2-4 mm. No bleeding or other procedure-related complications were observed at thoracoscopy, and the postoperative course was uncomplicated in all patients (procedure-related complications 0/3; 95% CI 0-70.8%) during a follow-up of 18 days. Given the small number of patients, these figures do not allow conclusions on success or complication rates. Patient characteristics and outcomes are summarized in Table 1. 

**Table 1:** Patient characteristics, procedural details and outcomes ICS: intercostal space​​​​​​; R0, microscopically tumour-free resection margin; VATS, video-assisted thoracoscopic surgery; Procedure time: first ultrasound contact to needle removal; Total added time: indocyanine green preparation, coil soaking, sterile set-up and puncture until start of one-lung; Coil–lesion and margin distances measured on the formalin-fixed specimen.

	Case 1	Case 2	Case 3
Age, years	52	62	67
Sex	Male	Female	Male
Smoking status	Active smoker	Never-smoker	Ex-smoker
Presentation	Chest pain; prior colon cancer	Incidental finding	Fever, cough for six weeks
Lesion location	Left upper lobe	Right lower lobe	Right upper lobe
Lesion size, mm	17	19	18
Depth below visceral pleura, mm	4	3	2
Skin-to-lesion distance (needle depth), mm	32	31	27
Intercoastal space	5^th^ ICS, mid-axillary line	8^th^ ICS, mid-axillary line	4^th^ ICS, mid-axillary line
Lesion appearance on CT	Solid	Solid	Solid
Coil-lesion distance, mm	3	4	2
Technical success	Yes	Yes	Yes
Procedure time, min	13	11	12
Number of needle passes	1	1	1
Total added time before resection, min	35	31	33
Lung sliding after puncture	Preserved	Preserved	Preserved
Interval to surgery	Immediate	Immediate	Immediate
Fluorescence identification	Yes	Yes	Yes
Resection margin	R0	R0	R0
Margin distance, mm	18	21	22
Surgical approach	Uniportal VATS	Biportal VATS	Uniportal VATS
Type of resection	Wedge resection	Segmentectomy	Segmentectomy
Histology	Metastatic adenocarcinoma (colorectal origin)	Squamous cell carcinoma	Squamous cell carcinoma
Postoperative course	Uncomplicated	Uncomplicated	Uncomplicated
Chest drain duration, days	2	3	3
Length of stay, days	4	6	4
Follow-up, post-op day	16	18	17

## Discussion

This case series illustrates that ICG-dyed coils can be placed percutaneously under transthoracic ultrasound guidance alone for intraoperative localization of selected peripheral lung tumors. To our knowledge, based on a PubMed/Embase search up to 1 October 2026 using the search terms “ICG”, “indocyanine green”, “fiducial” and “ICG-dyed coil”, this is the first report of this approach. Despite differing clinical presentations, comprising a suspected metastasis, an incidental finding, and a lesion detected during work-up of infectious symptoms, marker placement was successful, intraoperative localization with fluorescence was possible, and complete resection was achieved in all cases. However, these observations apply to a highly selected group. Only three of eight assessed patients from a total of 33 patients scheduled for marking during the study period had a lesion suitable for this approach.

A key observation is that lesion accessibility, rather than clinical presentation or histology, was the primary determinant of feasibility. All tumors were located within the subpleural surface and could be visualized sonographically. Aerated lung reflects ultrasound almost completely, so a lesion must either abut the pleura or be reachable through a sufficiently narrow rim of parenchyma to be seen. Lesions lying deeper in the parenchyma, or hidden behind the scapula, ribs, or mediastinal structures, remain unsuitable for this approach. We measured lesion distance and anticipated intercoastal trajectory in the coronal plane to reflect the lateral decubitus position in which the ultrasound examination was performed. In our experience, anticipating the actual procedural position during CT review is essential, as the shortest pleural distance on axial images may not correspond to an accessible intercoastal window.

The principal advantage of ultrasound guidance is the elimination of radiation exposure for both patient and staff, in contrast to CT-guided and cone-beam CT-guided techniques [17]. A further advantage is true real-time imaging. Whereas CT-guided placement proceeds stepwise with intermittent image acquisition, ultrasound allows continuous visualization of the needle, the lesion and the moving lung throughout the respiratory cycle. The needle time in our cases was short (median 12 minutes), but this figure covers only the interval from first ultrasound contact to needle removal and excludes coil soaking, sterile set-up and positioning. The median total added time before resection was 33 minutes. Because time definitions differ between studies and due to the small series, comparisons with published durations for CT-guided or bronchoscopic marking cannot be drawn.

The cases further suggest potential logistical benefits. Ultrasound is portable and widely available, so marking does not depend on CT or hybrid suite capacity and could in principle be performed at the bedside or in the operating room immediately before surgery. Moreover, the hyperechogenicity of the coil allows immediate sonographic confirmation of its position relative to the tumor, without additional imaging.

Because marking was performed immediately before resection and under the same anaesthesia, intraoperative alternatives must also be considered, including digital palpation, thoracoscopic intraoperative ultrasound or even percutaneous dye injection. The lesions in our series were solid, 17-19 mm in size and 2-4 mm below the visceral pleura, characteristics for which palpation or thoracoscopic ultrasound may also succeed. Nevertheless, none of the lesions was palpable at VATS, and in two of three patients no pleural change was visible. In uniportal VATS, the reduced incision length and single working port limit both palpation and instrument triangulation. In cases of dye injection, spillage and leaching may lead to incorrect marking and excessive tissue resection [9,18]. Thoracoscopic intraoperative ultrasound, although feasible, may be limited in cases of emphysema or incomplete lung collapse during surgery [19,20]. By comparison, an ICG-dyed coil provides a fixed, palpable, and fluorescent target that is independent of dye diffusion and, once the specimen is removed, confirms that the target is contained within it. These potential benefits must be weighed against the cost of the coil and the additional operating-room time. The added value of a coil over palpation or thoracoscopic ultrasound for such superficial lesions was not tested in this series and should be addressed in comparative studies. Likewise, R0 resection depends on surgical technique as well as on localization and cannot be attributed to the marker alone.

An important safety consideration is that any transthoracic marking technique involves pleural puncture and therefore carries a risk of pneumothorax and bleeding. CT-guided percutaneous localization is associated with relevant pneumothorax rates [9]. In our series, performing tumor marking immediately before surgical resection mitigated the clinical consequence of pneumothorax. Although lung sliding was preserved after needle removal in all patients, excluding an immediate pneumothorax, delayed pneumothorax could not be assessed because the lung was collapsed for resection immediately afterwards. No procedure-related complication was observed (0/3), but the upper limit of the 95% CI (70.8%) shows that three patients cannot establish the safety of the technique. This risk must be considered if marking is combined with delayed resection. It is conceivable that the short, subpleural needle path and real-time control inherent to ultrasound guidance may reduce the amount of aerated lung traversed and mitigate pneumothorax, but this remains to be demonstrated for the coil placement technique. For deeper lesions, bronchoscopic coil placement avoids pleural puncture altogether and has been associated with very low pneumothorax rates [14]. The ultrasound-guided approach should therefore be viewed as a complementary option within a broader localization strategy, in which the route is chosen according to lesion depth and position, rather than as a replacement for CT-guided or bronchoscopic marking.

This report has several limitations. It represents a small, retrospective, single-centre, descriptive series without a control group, which precludes any robust statement on success rates, complication rates or cost-effectiveness compared with established techniques. Patients were selected on the basis of favourable lesion location. Only three of eight assessed patients from a pool of 33 patients scheduled for marking at our centre were suitable, and this proportion may differ in other populations. Lesion size and morphology may also influence sonographic visibility; in particular, small or ground-glass lesions may be difficult to identify and were not specifically evaluated. All procedures were performed in a centre with experience in thoracic ultrasound and interventional pulmonology, which may limit generalizability. Marking was not combined with delayed resection; therefore, marker stability as well as pneumothorax risk over a long interval were not assessed, and the scheduling advantage of a persistent marker could not be evaluated. The added value over palpation or thoracoscopic ultrasound was also not tested. Finally, follow-up was limited to 18 days post operation; therefore, long-term outcomes were not assessed.

Despite these limitations, our findings suggest that ultrasound-guided placement of ICG-dyed coils is technically feasible and avoids radiation exposure in carefully selected patients with subpleural lung tumors. Future prospective studies should evaluate technical success, safety (including delayed pneumothorax and marker migration when marking is separated from resection) and learning curves in larger cohorts, define the lesion characteristics for which ultrasound guidance is suitable and compare outcomes with CT-guided and bronchoscopic approaches as well as with intraoperative palpation and thoracoscopic ultrasound

## Conclusions

In this small, selected series, ultrasound-guided percutaneous placement of ICG-dyed coils was technically feasible without radiation exposure and allowed fluorescence-guided localization of subpleural lung tumors at VATS, with R0 resection in all three patients. The technique was applicable to only a minority of assessed patients, and its safety, accuracy, and added value over intraoperative localization methods remain to be established. It may represent a useful complement to CT-guided and bronchoscopic marking that warrants prospective evaluation.
